# Influenza‐like illness in healthcare personnel at a paediatric referral hospital: Clinical picture and impact of the disease

**DOI:** 10.1111/irv.12553

**Published:** 2018-04-19

**Authors:** Almudena Laris González, Mónica Villa Guillén, Briceida López Martínez, Ana E. Gamiño Arroyo, Sarbelio Moreno Espinosa, Rodolfo Norberto Jiménez Juárez, José Luis Sánchez Huerta, Daniela de la Rosa Zamboni

**Affiliations:** ^1^ Hospital Infantil de México Federico Gómez Ciudad de México México

**Keywords:** healthcare personnel, influenza, morbidity

## Abstract

**Introduction:**

Healthcare personnel (HP) are frequently exposed to influenza and can be a source of transmission to patients and other workers, resulting in high‐cost outbreaks for healthcare institutions.

**Objectives:**

To analyse the presentation of HP with influenza‐like illness (ILI) and the differences between individuals with influenza confirmed by polymerase chain reaction (PCR) and those with a negative test. The secondary objective was to evaluate the duration of symptomatology and work absenteeism as well as the vaccination rate of HP at a paediatric referral hospital.

**Methods:**

A cross‐sectional, descriptive study was conducted at a paediatric referral hospital. Clinical and epidemiological data on HP with ILI were collected between January and April 2016. Nasopharyngeal swab for influenza PCR was obtained from one in every three workers with ILI. Telephone follow‐up was conducted to document duration of symptoms, complications and absenteeism.

**Results:**

A total of 164 ILI episodes were evaluated in 162 HP. A swab was obtained in 59 cases, and influenza was detected in 30 cases. The clinical picture of HP with confirmed influenza was similar to that of HP with a negative PCR. Arthralgia was more common in those with influenza (90% vs 58%), with a tendency towards statistical significance. No HP required hospitalization, and 78.5% were absent from work at least 1 day.

**Conclusions:**

Influenza causes significant morbidity and absenteeism among HP. Influenza infection was confirmed in only half of HP with an ILI on whom a PCR was performed, suggesting that other respiratory viruses can cause a similar pattern.

## 1 INTRODUCTION

Due to its unique antigenic characteristics, influenza virus infection occurs in annual outbreaks of varying magnitude and severity, leading to substantial morbidity and mortality, which disproportionately affects individuals with immunodeficiency, chronic diseases or those at the extremes of life. However, even people without identifiable risk factors can suffer complications and die from this illness.

Healthcare personnel (HP) are a vulnerable group due to frequent and close exposure to patients infected with influenza virus. In addition to the risks to one's own health, the infected worker can spread the virus to other patients, workers and their contacts in the community.^1^ Such transmission may result in outbreaks involving high costs for healthcare institutions.^2^ Annual vaccination of healthcare personnel is one of the main strategies for the prevention of transmission in this population, and a relationship has been documented between the vaccination coverage of healthcare personnel and a decrease in cases of nosocomial influenza,^3^ including in immunocompromised patients.^4^

During the 2015‐2016 season, influenza activity started to rise in January 2016. This increase in the number of cases was also detected at the Federico Gómez Children's Hospital of Mexico (Hospital Infantil de México Federico Gómez—HIMFG), National Health Institute, which annually treats more than 7000 low‐income patients from all over the country, especially from Mexico City and the State of Mexico (Estado de México).

The objective of this study was to analyse the presentation and evolution of HP with influenza‐like illness (ILI) in this setting as well as the clinical differences between those with influenza confirmed by polymerase chain reaction (PCR) of a nasopharyngeal swab and those who had a negative test.

The secondary objective was to evaluate the duration of the symptoms and work absenteeism caused by ILI as well as the vaccination rate of the HP at the HIMFG.

## 2 METHODOLOGY

This study is a cross‐sectional, descriptive study performed at the HIMFG, a national referral paediatric teaching hospital.

Following the increase in influenza cases detected in January 2016, staff with respiratory symptoms were invited to visit the Department of Hospital Epidemiology, and the promotion of respiratory etiquette measures was intensified through posters and vocal announcements.

HP who presented with ILI for diagnosis and treatment to the Department of Hospital Epidemiology were included in the study. Clinical and epidemiological information was collected from HP with ILI who were examined at the Department of Hospital Epidemiology between 27 January and 30 April 2016. ILI was defined as any acute‐onset episode, including at least one systemic symptom (fever ≥ 38°C, headache or myalgia) as well as a respiratory symptom (cough, odynophagia or respiratory distress).^5^ Nasopharyngeal swab for the detection of influenza virus was obtained from one in every three HP with ILI, plus a few other workers according to hospital needs.

Thirty‐five swab samples were processed by real‐time RT‐PCR (Applied Biosystems, Thermo Fisher Scientific corporation, USA) for the influenza A, AH1N1pdm and influenza B viruses, and 19 swabs were processed through the automated extraction of nucleic acids (MagNA Pure^**®**^ Compact, Roche) with detection of influenza A and B using an RT‐PCR system with microarray visualization (CLART^**®**^ PneumoVir, Genomica, Spain). Both techniques employed have a comparable diagnostic accuracy, with a specificity above 95%.^6^, ^7^

The HP were followed up by telephone using a previously designed survey to document the duration of the symptoms, the frequency of complications, work absenteeism and transmission to other people at home.

### 2.1 Statistical analysis

The results are expressed as measures of central tendency and dispersion; simple and cumulative frequencies were calculated. The group in which a nasopharyngeal swab was obtained was divided into subjects with confirmed influenza infection and subjects with a negative influenza test. Clinical and epidemiological characteristics were compared using Student's *t* test or the Mann‐Whitney U test for continuous variables and the chi‐squared test or Fisher's exact test for categorical variables.

Statistical analyses were performed using SPSS^®^ software version 20.

## 3 RESULTS

**Figure 1:**
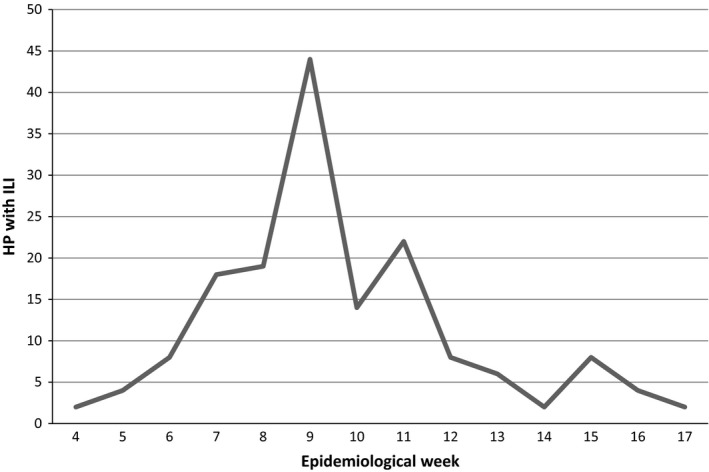
Number of healthcare personnel with influenza‐like illness assisted per week

One hundred and sixty‐two HP, who reported a total of 164 episodes of ILI, were examined (Figure 1). A nasopharyngeal swab was used to detect influenza virus in 59 (36%) of the 164 episodes. Table 1 shows the clinical and demographic characteristics of the subjects enrolled in the study. Almost three‐quarters of the staff who were examined for ILI were women, which represents a greater proportion than that found in the total population of hospital workers (74% vs 63.5%, *P* = .004).

**Table 1:** Clinical and demographic characteristics of HP with ILI evaluated in the Department of Hospital Epidemiology

Characteristic	All HP (N = 162 HP^a^, 164 episodes)	Swabbed HP (N = 59)	Not swabbed (N = 103 HP, 105 episodes)	*P* value
Female gender—No. (%)	121 (74.6)	44 (74.5)	77 (74.7)	.96
Age, median in years (interquartile range)	36.5 (29‐47.5)	33 (29‐44.7)	40 (29‐48)	.193
Occupation—No. (%)				
Nurse	38 (23.4)	12 (20.3)	26 (25.2)	.42
Affiliated physician	16 (9.8)	8 (13.5)	8 (7.7)	
Resident physician	47 (29)	21(35.6)	26(25.2)	
Others	61 (37.6)	18 (30.5)	43 (41.7)	
Comorbidities— No. (%)				
Obesity	22 (13.5)	8 (13.5)	14 (13.6)	.95
Diabetes mellitus	7 (4.3)	2 (3.4)	5 (4.8)	.68
Asthma	7 (4.3)	3 (5.1)	4 (3.9)	.70
Chronic obstructive	2 (1.2)	0 (0)	2 (1.9)	.53
Pulmonary disease (%)				
Immunocompromised	2 (1.2)	1 (1.7)	1 (1)	.6
Vaccination against seasonal influenza (%)	79 (48.7)	22 (37.2)	57 (55.3)	.04
Symptoms—No. (%)				
Cough	141 (86)	53 (89.8)	88 (83.8)	.28
Headache	139 (84.7)	53 (89.8)	86 (81.9)	.21
Chills	126 (76.8)	51 (86.4)	75 (71.4)	.016
Myalgia	125 (76.2)	49 (83)	76 (72.3)	.12
Fever >38°C	123 (75)	58 (98)	65 (61.9)	<.01
Arthralgia	112 (66.4)	44 (74.6)	68 (64.7)	.19
Odynophagia	96 (58.5)	37 (62.7)	59 (56.2)	.35
Chest pain	74 (45.1)	25 (42.4)	49 (46.6)	.59
Diarrhoea	32 (19.5)	11 (18.6)	21 (20)	.83
Sudden onset of symptoms	116 (70.7)	46 (77.9)	70 (66.6)	.10

a The characteristics of HP are presented as the total number and percentage of the 162 workers evaluated. The frequency of clinical symptoms is described for the 164 documented episodes of ILI.

**Figure 2:**
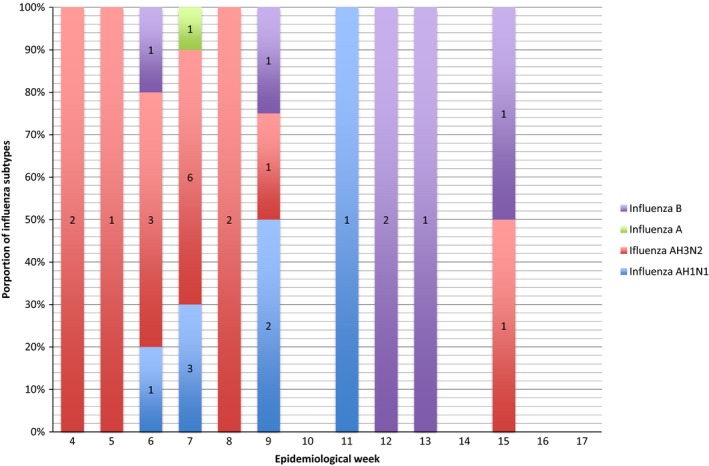
Proportion of influenza subtypes identified per week

Influenza virus was detected in 30 of the 59 nasopharyngeal swab specimens (50.8%), with the following distribution of subtypes: 16 influenza AH3N2, seven influenza AH1N1, six influenza B and one unidentified subtype of influenza A (Figure 2). Oseltamivir was given in 125 of the 164 episodes (76%), depending on the evolution time of the symptoms at the time of evaluation.

The frequencies of cardinal symptoms, including fever, headache, cough, myalgia and arthralgia, were compared in the subgroup in which nasopharyngeal swabs were obtained, and no differences were found between the groups with and without influenza (Table 2).

**Table 2:** Comparison of the clinical and epidemiological characteristics of influenza‐positive HP versus influenza‐negative HP

Characteristic	Positive PCR for influenza virus (N = 30)	Negative PCR for influenza virus (29)	*P* value
Female gender (%)	19 (63)	25 (86)	.044
Age, median in years (interquartile range)	32 (29‐46)	35 (29‐47)	.53
Occupation:			
Nurse	6	6	.3
Affiliated physician	6	2	
Resident physician	12	9	
Others	6	12	
Vaccination against seasonal influenza	11 (36%) AH1N1 4/7 (57%) AH3N2 6/16 (37%) A 0/1 (0%) B 1/6 (16%)	11 (38%)	.92
Most common symptoms (%)			
Fever	29 (96)	29 (100)	.32
Chills	28 (93)	23 (79)	.19
Headache	28 (93)	25 (86)	.36
Cough	25 (83)	28 (96)	.19
Odynophagia	18 (60)	19 (65)	.66
Myalgia	25 (83)	24 (83)	1.00
Arthralgia	27 (90)	17 (58)	.06
Diarrhoea	6 (20)	5 (17)	.78
Sudden onset of symptoms (%)	25 (83)	21 (72)	.19

Telephone follow‐up was performed for 79 of the 162 HP (49%) to assess the morbidity of the disease. None of the patients for which a follow‐up was performed had a fatal outcome or required hospitalization. Four patients (5.05%) required treatment with nebulizations, and ten patients (12.6%) reported secondary cases of influenza at home. For 62 cases (78.5%), no statistically significant difference in the duration of symptoms was found between patients with confirmed influenza and those with a negative viral panel (*P* > .05; Table 3).

**Table 3:** Duration of the symptoms and work absence in HP with ILI

	Number of HP who presented symptoms (%)	Duration in days (median)	Duration range in days (Interquartile range)
Fever	66/79 (83)	3	3‐5
Cough	69/79 (87)	4	3‐5
Headache	73/79 (92)	4	3‐5
Arthralgia	68/79 (86)	3	3‐5
Fatigue	65/79 (82)	3	3‐5
Work absenteeism	62/79 (78)	2	2‐3

**Figure 3:**
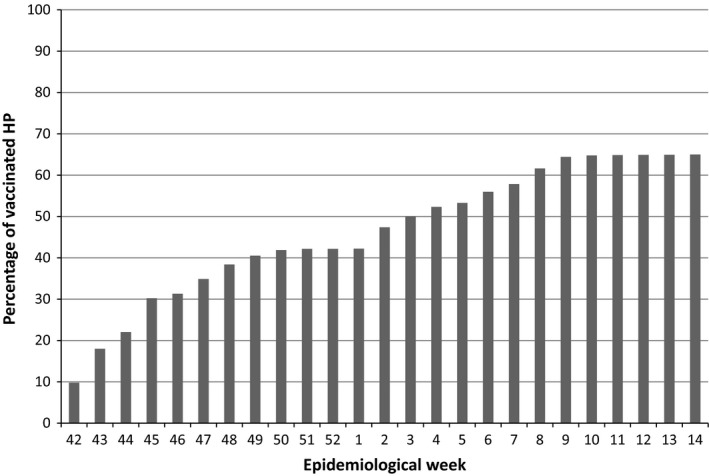
Percentage of healthcare personnel vaccinated against influenza by epidemiological week in the 2015‐2016 season

Regarding the immunization of the HP at the institution, the vaccine was started in the fourth week of October 2015, and by the end of December, 1246 doses had been administered to 42.2% of the total population of workers and resident physicians of the institution. Between January and April, 674 more doses were administered. A total of 1920 individuals were vaccinated, representing 65% of the aforementioned population (Figure 3).

## 4 DISCUSSION

Influenza is one of the most significant infections in HP due to its frequency in the winter season, the morbidity and absenteeism that it entails and the risk of transmission to patients susceptible to severe illness or even death.

The 2015‐2016 season was characterized by low influenza activity from October to December. However, beginning in the final week of January, a notable increase in cases of respiratory and influenza illness occurred both in patients and HP at the HIMFG, which is consistent with reports by the General Direction of Epidemiology (Dirección General de Epidemiología) at the national level.^8^

A high proportion of HP who sought ILI care were women, which is consistent with reports in the literature because women with ILI have been shown to be more likely to seek medical care than men.^9^ As shown in Table 1, there was a greater proportion of fever and chills among HP who were swabbed. As PCR was usually obtained from the first HP who consulted with ILI every day, a possible explanation for this finding is that HP with fever and chills were more likely to consult earlier in the day compared to workers without fever.

The most frequent symptoms in HP with influenza were fever, chills and headache, which were present in more than 90% of cases, followed by myalgia, arthralgia and cough, with a frequency greater than 80%. In contrast, in a study of HP in Chicago in 2014, only 51% of the participants presented with fever as a symptom.^10^ In our population, approximately one‐fifth of those assessed reported diarrhoea as a symptom. The prevalence of gastrointestinal symptoms has been reported in the literature to range from 2.8% to 30.9%, depending on the influenza subtype.^11^

It should be noted that no significant difference was observed in the clinical picture between individuals with documented influenza and those with a negative test, suggesting that other respiratory viruses may cause symptoms similar to those caused by the influenza virus. Arthralgia was the only clinical manifestation reported more frequently among individuals with influenza (90% vs. 58%), with a trend towards statistical significance (*P* = .06).

Among the HP for whom a telephone follow‐up was conducted, the average duration of systemic symptoms was four to five days. In 78% of the HP interviewed, an absence from their work in the hospital was required, with an average of two and a half days. According to data published by Tsai et al., the average work time lost by insured workers with ILI in the United States in the 2007‐2008 and 2008‐2009 seasons was approximately 24 hours per episode.^12^ Other authors who have studied the effect of influenza infection in the economically active population have reported an average of absenteeism between 1.5 and 4.9 days per episode of laboratory‐confirmed influenza.^13^ According to a study conducted at the University of Minnesota in 2006‐2007, vaccinated adults had a lower frequency of ILI episodes and work absenteeism.^14^

None of the interviewees required hospitalization, and no deaths were reported, although the population included individuals with risk factors for influenza complications, such as obesity (15%), asthma (5%) or diabetes mellitus (5%).^15^, ^16^

One of the most important prevention measures in healthcare personnel is the annual application of the influenza vaccine. However, low rates of vaccination have been detected in HP in different countries. In a region south of Italy, for example, only 25% of healthcare professionals were reported to have been immunized against influenza.^17^ At a referral medical centre in Afula, Israel, a vaccination coverage of 45% was documented in the 2013‐2014 season.^18^ In a third‐level hospital in South Carolina, the vaccination coverage of the workers was increased from 4% to 67%, resulting in a decrease in cases of nosocomial influenza among the patients.^3^ In a survey conducted in several paediatric hospitals in the United States, a vaccination rate of 43% was documented, with greater adherence among personnel working in high‐risk wards, such as oncology units.^19^

In the Children's Hospital of Mexico, influenza vaccination was started in the fourth week of October. By December, only 42% of the HP had been immunized, despite the availability of the vaccine for all staff as well as promotional campaigns. However, due to the increase in influenza cases in HP, acceptance of the vaccine increased, resulting in vaccination of 65% of HP.

Although in our population the proportions of vaccinated individuals were similar among workers with confirmed influenza and those with a negative test, the study was not designed to evaluate vaccine efficacy, and the sample size was not sufficient to obtain conclusions about this aspect.

Likewise, multifaceted strategies that include hospital policies for mandatory vaccination have been shown to be effective for increasing staff adherence to influenza vaccination.^4^

The limitations of the study include the fact that the detection of HP with ILI was passive and did not cover the entire influenza season, as it began in January. However, HP were constantly encouraged through posters, vocal announcements and personally to consult in case of ILI. Another data limitation of the study is the lack of follow‐up of half of the population because they could not be contacted to administer the questionnaire. However, the determination of other respiratory viruses using PCR of a nasopharyngeal swab would have been very useful to evaluate the aetiology of the ILI cases with a negative test result as well as the role of viral coinfections.
